# Falsified and substandard cardiovascular drugs in Africa: a need for continued monitoring strategies

**DOI:** 10.7189/jogh.09.020302

**Published:** 2019-12

**Authors:** Marie Antignac, Ibrahima Bara Diop, Diane Macquart de Terline, Melisande Bernard, Bernard Do, Méo Stéphane Ikama, Roland N'Guetta, Dadhi M Balde, Yessoufou Tchabi, Abdallahi Sidi Aly, Ibrahim Ali Toure, Patrick Zabsonre, Jean Marie F Damorou, Jean Laurent Takombe, Louise Boyer Chatenet, Kumar Narayanan, Eloi Marijon, Jean Philippe Empana, Xavier Jouven

**Affiliations:** 1 Department of Pharmacy, Saint-Antoine Hospital, AP-HP, Paris, France; 2 Paris Cardiovascular Research Centre, INSERM U970, Paris, France; 3 Department of Cardiology, University Hospital of Fann Dakar, Senegal; 4 University of Paris, Paris, France; 5 Department of Laboratories, Agence Générale des Equipements et Produits de Sante, AP-HP, Paris, France; 6 Faculty of Pharmacy, University of Paris Sud, Chatenay-Malabry, France; 7 Department of Cardiology, National University Hospital of Brazzaville, University of Marien NGOUABI, Brazzaville, Congo; 8 Department of Cardiology, Cardiology Institute of Abidjan, Abidjan, Côte d’Ivoire; 9 Department of Cardiology, University Hospital of Conakry, Guinea; 10 Unité de Soins, d'Enseignement et de Recherches en Cardiologie (USERC): National University Hospital of Cotonou, Benin; 11 Cardiology clinics, Nouakchott, Mauritania; 12 Department of Internal Medicine and Cardiology, University Hospital of Lamorde, University of Niamey, Niger; 13 Department of Cardiology, National Sanou Souro de Bobo-Dioulasso Hospital, Ouagadougou, Burkina Faso; 14 Department of Cardiology, Hospital of Lomé, Togo; 15 Department of Internal Medicine, General Hospital of Kinshasa, the Democratic Republic of Congo; 16 Department of Cardiology, Georges Pompidou European Hospital, AP-HP, Paris, France; 17 Maxcure Hospitals, Hyderabad, India

**Figure:**
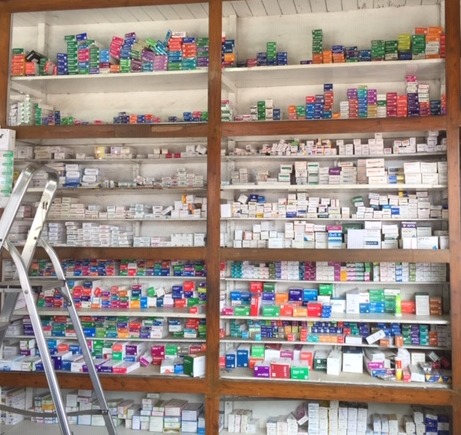
Photo: from the collection of Marie Antignac (used with permission).

Since 20 years, Africa has witnessed an exponential rise in cardiovascular risk factors. Today, if all people with hypertension in sub-Saharan Africa were treated effectively, about 250 000 deaths would be prevented annually [1].

Substandard/falsified antihypertensive medicines pose a serious health hazard and result in significant morbidity and mortality [2]. Drug quality assessments have mainly focused so far on anti-infective drugs [3]; we recently published data on the quality of cardiovascular drugs in Africa (SEVEN study) for the first time [4]. A significant proportion of drugs were found to be of poor quality. Considering the extent and scope of the falsified/substandard drugs problem shown in this report, consistent national and international scrutiny is required as well as increased public awareness and ongoing monitoring strategies are crucial.

## BACKGROUND

Appropriate drug therapy forms a major pillar of cardiovascular (CV) risk reduction and many CV deaths could be avoided with an optimized use of antihypertensives, statins and anticoagulants among patients in limited-resource countries [5].

Availability, affordability, acceptability, accessibility, and quality [5] are the 5 described dimensions of access to medications in low and middle-income countries. Data are particularly scarce regarding the quality of CV drugs. Despite investments to strengthen enforcement of quality standards in developing countries, the seizure of counterfeit pills continues to make headlines [6].

## RISKS WITH SUBSTANDARD / COUNTERFEIT MEDICINES

Medicines are an essential building block of a functioning health care system. Poor quality drugs used in cardiology may cause treatment failure that leads to increased mortality in short (beta blockers, anticoagulants) or long (statin) terms. Major instances involving falsified cardiovascular drugs are rarely reported, but can have tragic consequences, as exemplified by the 120 Pakistanis who died after taking a carelessly made batch of isosorbide mononitrate [2]. Furthermore the potential variability of quality of drugs might cause major risks to patients. For instance, dosage adjustments of vitamin K antagonists (VKA) are delicate, variations of quality could further lead to incorrect titrations. Additionally, loss of confidence in health care system and drug regulatory authorities is inevitable in areas where drug quality is perceived as being poor.

## SUBSTANDARD VS COUNTERFEIT DRUGS

In high income countries, the issue of poor quality drugs is approached from a single dimension: the fight against counterfeit medicines which is reported more frequently in newspapers and online resources [7-10] (http://psi-inc.org/counterfeitSituation.cfm) than in formal medical literature [3]. Reported cases often concern expensive medicines (bevacizumab), dramatic events (such as death due to treatment of erectile dysfunction with a counterfeit drug) [11], or online purchase fraud. Therefore, identification of counterfeit drugs takes primary importance.

However, in developing countries, the whole supply chain is being of poor quality, of which falsified medicines represent only one type. A more widespread problem may be that of *substandard* medicines that “are genuine medicines produced by legitimate manufacturers that do not meet the quality specifications that the producer says they meet. For example, they may contain less (or more) active ingredient than written on the package [12] quality can result in: increased, reduced concentration or absence of active ingredient, reduced stability of treatment pack (degraded medicines may result from inappropriate exposure of good-quality medicines to light, heat and humidity), impurities, wrong, altered or unknown ingredients and inappropriate packaging [3].

In 2011, the World Health Organization (WHO) incorporated counterfeit and substandard medicines under the new term “substandard/spurious/falsely-labeled/falsified/counterfeit medical products” (SSFFC). In 2017, WHO standardizes the terms describing the different mechanisms resulting in poor-quality medicines: Counterfeit: the unauthorized use of a trademark to identify a medicine; Falsified: a medicine in which there is an intention to deceive; Substandard: a product that fails to meet pharmacopeial standards, and Unregistered: product that lacks market authorization from the national regulatory authorities [13]. In the same way, a recent review on the quality of antimicrobial agents choose not to differentiate the different categories of poor quality and used the term “substandard/counterfeit” [3].

## EXISTING MEASURES TO COMBAT POOR QUALITY DRUGS

International public health organizations are at the forefront of developing measures to fight against counterfeiting and poor quality drugs [14]. The WHO pre-qualification is a systematic process to determine the capacity of a manufacturer to produce consistent quality products in accordance with international standards and WHO specifications. It provides the list of those meeting WHO standards to countries and procurement agencies to promote the purchase of good quality medicines. A recent review concluded that policymakers and stakeholders would benefit from registration and WHO-prequalification of drugs and may also consider other multifaceted interventions [15]. Presently, no manufacturing facilities in western Africa [16] are pre-qualified by WHO. This initiative primarily concerns medicines for HIV/AIDS, malaria and tuberculosis meanwhile, concrete actions to improve the quality of drugs used in non-communicable diseases still remain modest. Despite WHO efforts, according to The Pharmaceutical Security Institute data, instances of counterfeit medicines increased dramatically from 196 incidents in 2002 to 2193 in 2013 [10] and involved 317 different drugs including cardiovascular drugs (8% increase).

## OUR MULTIDISCIPLINARY (LABORATORY/PHYSICIAN/PHARMACIST), MULTINATIONAL (AFRICA/FRANCE) TEAM: AN INNOVATIVE COLLABORATION TO HELP FIGHT POOR QUALITY DRUGS

Our novel initiative is based on an innovative multinational and multidisciplinary collaboration (cardiologists, epidemiologists, pharmacists from France and Africa) which has extensive prior research experience in the field of Rheumatic heart disease [17] and sickle cell disease.

The high proportion of poor quality drugs recently reported from 10 African countries [4] demonstrates the need for public awareness and ongoing monitoring strategies. In this context, availability of laboratories to test drug samples with a standardized and validated method is of crucial importance.

The dosage method, specially developed for the SEVEN study by a certified public laboratory in France [18] is a validated reversed-phase liquid chromatography with tandem mass spectrometry method to accurately quantify the amount of active ingredient. This kind of method was described [3] as a more advanced level, and bestows several advantages compared to colorimetric techniques, visual inspection, dissolution assays, spectroscopy methods and infrared. It allows accurate identification of the active ingredient and determination of its exact quantity, thereby assessing drug quality according to the specifications established by international pharmacopeia. As counterfeiting approaches have become more and more sophisticated, common techniques to assess drug quality need to be backed up by more specific and informative methods. Thus far, the cheapest methods have generally been preferred. For instance, the Global Pharma Health Fund e.V. Minilab (http://www.gphf.org/web/en/minilab/index.htm) used semiquantitative thin-layer chromatography and disintegration tests on each sample to determine the presence and relative concentration of active ingredient. In a study exploring antimalarial drug quality assessment between two periods (2007 and 2010), authors showed that drug quality is probably improving, but also raised concerns that some counterfeit producers may be adapting products to pass Minilab tests by including small amounts of active pharmaceutical ingredient to pass basic analyses, such as color dye tests, which only detect the presence of active principal ingredient, not the quantity. In the SEVEN study too, all samples contained the expected active ingredient. Therefore, it follows that to accurately detect poor-quality drugs, standardized methods with good quality control, supporting technology, and well-trained personnel are essential.

To plan our study and communicate the results, we liaised with government and/or administration in several countries to enlist their support and cooperation. However, even though we encountered motivated people, several obstacles remain in sub-Saharan countries. First, frequent administrative changes in the health sector hamper continuity of policy and rational planning. Second, conflicting priorities between different ministries (Health and Trade, for example) result in lack of harmony: trade-related agreements and legislation could potentially authorize the production and sale of medical products that health authorities would otherwise refuse. Finally, concrete legislation on authorized vs non-authorized medical products is lacking in most sub-Saharan countries.

One potential solution to overcome these obstacles is to set up controls at the level of the consumer or end user, ie, directly on street markets and pharmacies by regular and random samplings. These controls will be considered as objective and valid only if they are performed outside the country to avoid conflicts of interest with in-charge authorities.

The French laboratory will continue to offer assessment of the quality of the seven cardiac drugs tested in the SEVEN study for the next five years. African physicians, thanks to the multidisciplinary network, are able to directly send us collected drug samples for which poor quality is suspected after other causes of lack of efficacy are ruled out such as incorrect drug regimen(according to international recommendations), patient’s adherence etc. The results of quality analysis are communicated personally to the sender physician. This initiative can have several favorable consequences for patients and clinicians; it could help to understand the reason for poor efficacy of anti-hypertensive drugs in some cases. When the quality of medicines is systematically assessed in available legal (pharmacy) and illegal (street-markets) outlets, areas of risk with especially high proportion of substandard drugs can be identified from the public health authority point of view.

The SEVEN study authors initially presented this methodology to all the councils of pharmacists, in 2014 during the general assembly of CIOPF [19], and they communicated the proportion of poor quality drugs by country to the Council of Pharmacists from all participating countries.

In conclusion, the SEVEN study initiative demonstrates the feasibility of taking steps towards combating substandard drugs through ongoing monitoring strategies. We propose to help the setting up of more such independent free testing but further steps to ensure corrective action by individual national authorities requires increasing international surveillance on this important issue and sustained public education. A multidisciplinary multinational collaborative team is needed to organize and promote this effort together with concrete international political will.
